# Anti-Sa antibodies and antibodies against cyclic citrullinated peptide are not equivalent as predictors of severe outcomes in patients with recent-onset polyarthritis

**DOI:** 10.1186/ar1719

**Published:** 2005-03-17

**Authors:** Gilles Boire, Pierre Cossette, Artur J de Brum-Fernandes, Patrick Liang, Théophile Niyonsenga, Zhijie J Zhou, Nathalie Carrier, Claude Daniel, Henri-A Ménard

**Affiliations:** 1 Department of Medicine, Division of Rheumatology, University of Sherbrooke, Sherbrooke, Quebec, Canada; 2 Department of Medicine, Division of Internal Medicine, University of Sherbrooke, Sherbrooke, Quebec, Canada; 3 Centre de recherches cliniques, Centre hospitalier universitaire de Sherbrooke (CHUS), Sherbrooke, Québec, Canada; 4 Division of Rheumatology, Department of Medicine, McGill University Health Centre, Montréal, Quebec, Canada; 5 Laboratoire d'histocompatibilité, Université du Québec, INRS-Institut Armand-Frappier, Laval, Québec, Canada

## Abstract

The prognostic value of two antibodies targeting citrullinated antigens, anti-Sa and anti-cyclic citrullinated peptide (CCP), present at inclusion, was evaluated prospectively in a cohort of 165 consecutive patients with recent-onset or early polyarthritis (EPA) followed for up to 30 months. Patients were treated according to current Good Clinical Practice standards. Predefined outcomes were severe arthritis and persistent arthritis. At inclusion, a median of 3 months after disease onset, 133 (81%) patients fulfilled at least four American College of Rheumatology criteria for rheumatoid arthritis and 30 (18%) had erosive changes on radiographs of hands and feet. Disease-modifying anti-rheumatic drugs were used in close to 80% of the patients at 30 months. Joint damage increased linearly over time, whereas disease activity declined markedly and remained low at each follow-up. Autoantibodies were identified in 76 (46%) patients: rheumatoid factor (RF) in 68 (41%), anti-CCP in 53 (33%), and anti-Sa in 46 (28%). All three antibodies were correlated, but anti-Sa antibodies best predicted severity at 18 and 30 months. RF and anti-CCP performed less well. For both outcomes, anti-Sa alone performed better than any combination of antibodies. The presence of any autoantibody identified about 50 to 60% of the patients with poor outcomes. In multivariate analysis, anti-Sa (odds ratio (OR) 8.83), the presence of erosions at inclusion (OR 3.47) and increasing age (OR 1.06/year) were significantly associated with severity, whereas RF and anti-CCP were not significant predictors. Persistent arthritis was present in up to 84% of patients; autoantibodies were specific but poorly sensitive predictors of this outcome. We conclude that assays for antibodies against citrullinated antigens differ in their ability to predict poorer outcomes in patients with EPA. In our EPA cohort treated in accordance with current standards, detection of anti-Sa but not of RF or anti-CCP antibodies, in combination with clinical and radiological variables present at the first encounter, allowed the identification of a subgroup of EPA patients suffering more rapid and more severe joint damage over 30 months.

## Introduction

Rheumatoid arthritis (RA) is a chronic inflammatory arthritis that frequently starts at the peak of productive life and is a major cause of invalidity, morbidity, and premature mortality [1]. RA is characterized by an early inflammatory stage that is frequently responsive to disease-modifying anti-rheumatic drugs (DMARDs) [2]. At a temporally undefined later stage, the RA process evolves towards pannus formation responsible for the joint destruction. Once established, pannus may progress on its own, independently of the apparent response to DMARDs. No available treatment can reverse significant joint damage. These observations gave rise to the notion of a therapeutic 'window of opportunity' during which the rheumatoid process would be more likely to be stopped or retarded [2,3]. This notion is supported by the fact that aggressive treatment of early RA decreases both mortality and long-term invalidity and can increase the rate of long-term remission [4-8]. The identification, early into disease, of those patients likely to evolve rapidly to pannus formation and destructive/disabling arthritis would allow the most cost-effective use of expensive treatments and, reciprocally, would minimize unnecessary exposure of spontaneously remitting patients to the risks of aggressive treatments.

Sufficiently specific and sensitive prognostic markers that could be used with confidence in the individual patient with early or recent-onset polyarthritis (EPA) and recent-onset RA are still lacking [9-11]. For example, even within patients fulfilling the 1987 revised classification criteria for RA of the American College of Rheumatology (ACR; formerly the American Rheumatism Association) [12], chronic arthritis presents wide variations in response to treatments, degree of inflammation, and potential for joint destruction and functional impairment [13]. Classification criteria have even more limited value in predicting outcomes of patients with recent-onset polyarthritis [14]. A second challenge is the frequent occurrence of spontaneous remission in early polyarthritis present for up to 3 to 6 months. This relatively benign evolution is well documented, both in population-based studies [15,16] and in cohort studies of patients with polyarthritis of recent onset [17,18]. As a consequence, clinicians frequently adopt a watch-and-see attitude with patients during the first months of disease, delaying treatment with irreversible detrimental consequences [6].

Recently, antibodies targeting determinants resulting from the deimination of peptidylarginine to peptidylcitrulline residues have been described in the serum of patients with RA [19]. These include antibodies targeting cyclic citrullinated peptide (CCP) [20] and antibodies targeting *in vivo *citrullinated proteins, such as anti-keratin antibodies, antiperinuclear factor, anti-citrullinated (pro)filaggrin and anti-Sa/citrullinated vimentin [21,22]. The specificity of these antibodies for established RA, either rheumatoid factor (RF)-positive or RF-negative, and their presence in patient sera in the preclinical and early clinical phases of disease have been documented [23,24]. The serum of most patients with diseases other than RA, either RF-positive or RF-negative, does not contain antibodies against citrullinated peptides or proteins [20]. Assays to detect these anti-citrullinated peptides/proteins might therefore be useful to predict poor outcomes in patients with early polyarthritis [25-27], although their added value relative to RF was found to be modest or even controversial [28-31].

The purpose of our study was to assess the prognostic role of anti-Sa and of anti-CCP antibodies, relative to RF, in patients with polyarthritis evaluated and treated early after the onset of disease, irrespective of the fulfillment or not of diagnostic criteria for a specific disease entity. We now present an analysis of our cohort at 30 months of follow-up.

## Methods

### Patients

Consecutive adult patients with synovitis affecting at least three joints for at least 1 month and less than 12 months and evaluated at the Centre hospitalier universitaire de Sherbrooke (CHUS) were asked to participate to the study. The CHUS is the single regional rheumatology referral center for about 400,000 people, and is the site of practice of six of the seven rheumatologists in the area. Patients with bacterial or crystal-induced arthritis, patients with a defined connective tissue disease (looked for both clinically and by autoantibody testing [32]), patients with systemic vasculitis according to ACR criteria [33], and those who declined or were unable to consent were excluded. Failure to fulfill ACR criteria for RA [12], the presence of skin or nail lesions typical of psoriasis or psoriasis-like lesions, inflammatory bowel disease, clinical features suggestive of a spondylarthropathy [34], signs or symptoms suggestive of a connective tissue disease without fulfillment of specific ACR criteria, and the presence of positive HLA-B27 were not reasons for exclusion. Most of the included patients were regularly followed by rheumatologists and treated with the current approach of early and intensive treatment with DMARDs [2,35]. Patients, rheumatologists, coordinating nurses and treating physicians remained blinded to the patients' HLA-DR, anti-CCP and anti-Sa status. Serum and DNA materials were coded to ensure confidentiality and blinding of investigators. All patients gave their informed consent and the ethics review board of the CHUS approved the study.

### Disease variables

A rheumatologist completed a structured physical examination, including 68 tender and 66 swollen-joint count assessments and identification of extra-articular manifestations. A trained nurse coordinator performed a structured interview at the inclusion visit and at each of the follow-up visits scheduled at 18, 30, 42 and 60 months after the onset of inflammatory arthritis. The time of onset of arthritis was assumed to be the month during which the patient indicated that joint symptoms/signs had appeared or, in patients with previous musculoskeletal complaints (such as osteoarthritis), at the time that the signs or symptoms of inflammatory arthritis appeared, additive to the previous signs and symptoms. Scheduling the follow-up evaluations relative to the onset of symptoms rather than to the establishment of diagnosis sought to ensure that the cohort was more homogeneous in disease duration on follow-up.

Variables assessed included demographics, tender and swollen joint counts, duration of morning stiffness, use of DMARDs and corticosteroids at and between each visit, modified Health Assessment Questionnaire (M-HAQ) [36], erythrocyte sedimentation rate (according to the Wintrobe method), serum C-reactive protein (CRP; lower limit of detection 3.0 mg/L, upper normal limit 8.0 mg/L), serum RF (latex agglutination, RapiTex RF; Dade Behring Inc, Newark, DE, USA; threshold for positive results set at 40 IU/ml on the basis of clinical experience and confirmed by its optimal prognostic value), genomic typing of the HLA-DR (see below), measure of anti-Sa antibodies (see below) and anti-CCP antibodies (QuantaLite™; Inova Diagnostics, San Diego, CA, USA; in accordance with the manufacturer's recommendations). Standardized radiographs of the hands and feet were obtained at inclusion and at each scheduled assessment. Joint space narrowing and erosions were scored by the Sharp–van der Heijde (SvH) method, with a maximum score of 448 [37]. All radiographs were read in pairs in a known time sequence by a trained reviewer who was blinded to the corresponding clinical information. The smallest detectable difference was shown to be 5 units; this was assumed to be identical to the minimal clinically important difference [38,39]. Functional status was determined at each visit by using validated French-Canadian or English versions of the M-HAQ with a range of 0 to 3 (good functional status to maximal disablement). Disease Activity Score using 28 joints and 3 variables (tender and swollen joint counts and CRP; DAS28-3) was calculated with the appropriate formulas  [40]. A DAS28-3 score below 2.6 defined clinical remission [41,42], whereas a score above 3.2 indicated more than mild disease.

### Predefined outcomes

Persistent arthritis was defined as the presence of at least one joint with synovitis and/or the current use of DMARDs or at least 10 mg of prednisone equivalent per day [25]. Fulfillment of RA criteria required at least four ACR criteria for RA. Severity required an M-HAQ score of at least 1.0 and/or belonging to the upper third of the SvH radiological score.

### Genomic PCR typing at the HLA-DR and HLA-DQ

Genomic DNA was extracted from leukocytes present in 2.5 ml of EDTA-treated whole blood using Wizard DNA extraction kit (Promega Corporation, Madison, WI, USA), then stored at 4°C. Genomic typing was performed by using PCR with sequence-specific primers specific for HLA class II molecules [43,44]. Primer sets for low-resolution typing of HLA-DR and HLA-DQ antigens, and high-resolution typing of DRB1*01, DRB1*04, DRB1*14 and DQB1 loci were from Pel-Freez Clinical Systems (Brown Deer, WI, USA). After amplification, PCR products (10 μl) were resolved in a 2% agarose gel containing ethidium bromide and detected by ultraviolet transillumination. Amplification patterns were interpreted in accordance with the manufacturer's instructions. HLA-DRB1 alleles encoding the amino acid sequences QRRAA (DRB1*0101, DRB1*0102, DRB1*0105, DRB1*0404, DRB1*0405 and DRB1*0408), QKRAA (DRB1*0401 and DRB1*0409) and RRRAA (DRB1*1001) were considered to encode the shared epitope.

### Anti-Sa/citrullinated vimentin

The Sa antigen was first detected in human spleen/placenta extracts by western blotting (WB) [21] and subsequently identified as citrullinated vimentin [22]. Most citrullinated arginine-rich proteins are adequate but not absolutely equivalent reagents for the detection of antibodies against citrullinated epitopes, as discussed in [22]. Because access to human tissues and consistent extraction of the Sa antigen from tissues proved problematic, and commercial human vimentin is very expensive, we developed standardized and less expensive assays. We screened several cell lines and identified the ECV 304 endothelial cell line as rich in both vimentin and peptidylarginine deiminases (PADIs). Auto-citrullinated extracts from ECV 304 cells (see below) paralleled human placenta extracts for anti-Sa detection in WB. We then used this ECV 304-based WB assay to monitor the performance of an in-house enzyme-linked immunosorbent assay (ELISA), designed with *in vitro *citrullinated bovine myelin basic protein (MBP). MBP and vimentin have similarly high proportions of arginine. Most positive results in MBP-ELISA correlated with positive anti-Sa results on the original WB method using human placenta.

#### Anti-Sa WB assay

ECV 304 cells were cultured at 37°C under 5% CO_2 _in IMDM (Sigma Chemical Co., St Louis, MO, USA) enriched with 10% fetal bovine serum containing 3.024 g/L sodium bicarbonate (pH 7.2). At confluence, cells were trypsinized, washed, incubated for 5 min on ice in 1 ml of buffer containing 40 mM Tris-HCl pH 7.5, 1 mM EDTA pH 8.0, 0.150 NaCl, and mechanically detached. The resultant cell pellet was submitted to three freeze-thaw cycles, and the supernatant was clarified by centrifugation. The concentration of protein was measured by the Bradford assay (Bio-Rad), and the extracts were stored at -80°C. In preparation for WB, the extracts (2.5 μg per tested serum) were auto-citrullinated for 3 hours at 37°C in 50 mM citrullination buffer (Tris-HCl pH 7.5, 10 mM CaCl_2_, 10 mM dithiothreitol). The reaction was stopped with EDTA (100 mM final concentration). An equal volume of 2× WB loading buffer was added to the citrullinated extract for long-term storage in aliquots at -20°C. WB was performed as described [21], with ECV 304 native and citrullinated extracts being run in parallel. Sera were tested in duplicate at a dilution of 1:100 and results are expressed as either positive or negative.

#### Anti-Sa ELISA

Bovine MBP (Sigma) was citrullinated *in vitro *by incubation for 3 hours at 37°C in citrullination buffer containing 0.2 U rabbit PADI 2 (Sigma) per μg of MBP. Microtiter plates (96 wells; Nunc Maxisorp, WWR International Ltd., Mississauga, ON, Canada) were coated overnight at 4°C with native or citrullinated bovine MBP at 1 μg per well. The plates were blocked for 1 hour at room temperature (20 to 25°C) with PBS containing 1% (w/v) BSA, then washed three times with PBS containing 0.05% Tween 20 (PBST). The plates were incubated for 1 hour at room temperature with 100 μl per well of human serum diluted 1:300 in PBS containing 1% (w/v) BSA (PBS-BSA), and then washed three times with PBST. Bound human IgG was detected with a goat anti-human IgG, alkaline phosphatase-labeled conjugate (Jackson ImmunoResearch Laboratories Inc., West Grove, PA, USA) diluted 1:1000 in PBS-BSA. The reaction was detected with 1 mg/ml *p*-nitrophenyl phosphate (Sigma) in substrate buffer (10% diethanolamine, 0.5 mM MgCl_2_, pH 9.8) for 20 min at room temperature. The absorbance, *A*, was read at 405 nm. Each sample was run in duplicate and in parallel on native MBP (N), on citrullinated MBP (C), and on BSA (B). Results were given by the equation *A*_405 _= *A*_C _– *A*_N_. A positive test was defined as *A*_405 _> 0.2. A known positive control serum was used on each plate for reagent quality control. The BSA control was used to detect spurious intrinsic individual serum stickiness for which some RA sera are notorious. When a serum gave high *A*_405 _when used on BSA alone, a positive anti-Sa ELISA result had to be confirmed on WB to be reported as positive. All the anti-Sa assays described here were developed and performed on coded sera at the McGill University Autoimmune Research Laboratory.

### Statistical analysis

The principal independent variables were anti-Sa (MBP-ELISA), anti-CCP (positive if more than 20 U/ml), and RF (positive if at least 40 IU/ml) antibodies. The degree of association between anti-Sa and anti-CCP assays was measured by using the phi coefficient. Sensitivity, specificity and positive likelihood ratio (LR) were calculated for each of the three outcome measures at 18 and 30 months into disease and for each of the 13 following putative prognostic markers present at the inclusion visit: anti-Sa, anti-CCP, RF, sex, age (in years), duration of disease (in months), duration of morning stiffness (positive when at least 1 hour), symmetry of joint swelling, number of joints with synovitis, M-HAQ score (positive when at least 1.0), RA-associated HLA-DRB1 alleles ('shared epitope'), CRP levels (positive when higher than 8.0 mg/L), and an erosion component of the SvH score of at least 5. The predictive ability of anti-Sa, anti-CCP, and RF in the early identification of patients with each outcome was also studied by using the area under a receiver operating characteristics curve. Confidence intervals were calculated for these estimates. After these estimations, a logistic regression approach was used to estimate the independent contribution of RF, anti-Sa, and anti-CCP present at inclusion to predict the development of outcomes at 30 months. Each autoantibody was evaluated separately and in combination, after inclusion in the multivariate statistical model of prognostic factors listed above. A final statistical model was constructed with the stepwise logistic regression approach. By using the variables included in the final model, with and without the inclusion of anti-Sa and anti-CCP antibodies, the odds ratio (OR) was calculated to estimate the contribution of each marker adjusted for the other markers in the prediction of the severity outcome.

## Results

### Patient characteristics at study entry

Up to May 2004, 260 patients agreed to participate and were included. Eleven additional patients declined to participate or had very incomplete inclusion data and were excluded. At this time, 165 patients had reached the 30-month evaluation mark. Of these patients,5 had died and 11 failed to come to their scheduled appointment. We therefore report on the 149 patients (retention rate 90.3%) with a complete 30-month assessment.

The baseline characteristics at entry of the 165 patients are summarized in Table 1. The 16 patients who died or who were lost to follow-up did not differ significantly on any of the inclusion variables from the 149 who were followed up (data not shown). Men (42.4% of our patients) were slightly older than women (61.7 versus 58.0 years). These patients had a disease of short duration (mean 4.4 months, median 3 months). Nevertheless, at inclusion, 80.6% of the patients already fulfilled at least four ACR criteria for RA, 47% were functionally disabled (M-HAQ at least 1.0), and 18% had significant erosive changes (SvH erosion score of 5 or more).

The prevalence of autoantibodies was low. RF was found in 68 (41.2%) patients, anti-CCP in 53 (33.1%), and anti-Sa in 46 (28.0%). As reported previously [20,29], the presence of anti-CCP, RF, and anti-Sa was moderately to highly correlated (Kendall's tau-b > 0.6 for all comparisons). A total of 76 sera contained either one of RF, anti-CCP, or anti-Sa antibodies, and 35 sera contained all three. RF and anti-CCP co-existed in 46 patients, RF and anti-Sa in 41, and anti-Sa and anti-CCP in 39. This means that, despite a good correlation between assays, 13 (19.1%) of the 68 sera with RF did not contain antibodies against citrullinated antigens, CCP or Sa. Interestingly, the degree of association between anti-Sa and anti-CCP (titer higher than 21 U/ml; phi coefficient 0.6881) was very similar to the degree of association between anti-Sa and high titer anti-CCP only (titer higher than 100 U/ml; phi coefficient 0.6339). This absence of variation of the degree of association with increasing titers suggests that anti-Sa and anti-CCP assays differ qualitatively. The shared epitope at the HLA DRB1 locus was present in 77 (47%) of all patients, and in 62 (47%) of those fulfilling criteria for RA at inclusion. Twenty-two patients (13.4%) carried two shared epitope alleles.

### Evolution of the clinical status from inclusion to the 18-month and 30-month evaluations

**Figure 1:**
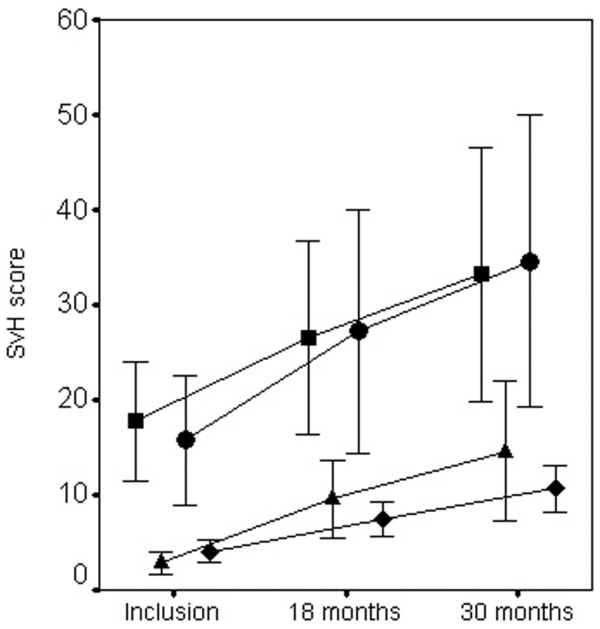
Total Sharp–van der Heijde (SvH) score at inclusion and at 18 and 30 months. Mean values and 95% confidence limits are illustrated. The four curves represent groups of patients at inclusion who had an erosion SvH score of at least 5 and positive anti-Sa (15, 14, and 14 patients at each visit) (squares), an erosion SvH score of at least 5 and negative anti-Sa (15, 13, and 13 patients at each visit) (circles), an erosion SvH score of 4 or less and positive anti-Sa (32, 30, and 30 patients at each visit) (triangles), and an erosion SvH score of 4 or less and negative anti-Sa (103, 97, and 92 patients at each visit) (diamonds).

Upon follow-up, the clinical status of the patients improved markedly (Table 2). The DAS28-3, the M-HAQ, and the swollen joint counts all significantly decreased. Despite this apparent control of clinical disease activity relative to inclusion, the radiological SvH score increased steadily at each follow-up, both in the total score and in its erosion component (Fig. 1). A high proportion (77.9%) of patients was still being actively treated with DMARDs at 30 months. Interestingly, despite blinding of their treating physicians, the patients with anti-Sa and anti-CCP were treated as intensively as RF-positive patients. Most treated patients were on 15 to 25 mg/week methotrexate, with about two-thirds (77 of 116) on combination therapies. Despite this liberal use of DMARDs, nine patients with at least one swollen joint were not being treated with DMARDs at 30 months. Those untreated patients represented 13% of patients with synovitis at this follow-up. According to our definition (see above and similar to that in [25]), persistent arthritis was said to be present when swelling was detectable in at least one joint and/or when at least one DMARD or at least a moderate dose of corticosteroids was used at the time of follow-up. Thus, 88% and 84% of the patients were considered as having persistent arthritis at 18 and 30 months, indicating that long-term spontaneous remission was unusual in our EPA cohort, as reported previously in [45]. Close to half (namely 53 of 137 and 58 of 125, at 18 and 30 months, respectively) of the patients labeled 'persistent arthritis' had no swollen joints at each follow-up. The good clinical control of disease in our patients was also evident when using the DAS28-3 score: 76 (48.7%) had a score of less than 2.6 ('remission' [41,42]) at 18 months, and 82 (55.0%) at 30 months. Thus, more than half of our initial cohort was in remission as defined by DAS28-3 at 30 months, arguably the highest proportion of remission in a cohort of patients with EPA [6].

Severe arthritis was present in 56 (38%) patients at 30 months. Our inclusive definition of severity (upper third of SvH score and/or M-HAQ ≥ 1.0) did not make any *a priori *assumption about the rate of damage progression in our patients, as this should be highly dependent on the characteristics of the patients included and (possibly) the treatments used. The threshold for the upper third of SvH score was calculated as a total score of more than 10 at 18 months and more than 15 at 30 months, that is, about 2% and 3.5%, respectively, of the maximal SvH score. Not surprisingly, functional impairment was the criterion most determinant for selection in the group with severe disease at inclusion, whereas radiological damage became the major reason for selection during follow-up.

Only 28 (18.79%) patients still fulfilled at least four ACR criteria for RA at 30 months. At inclusion or on follow-up, 20 patients fulfilled criteria for inflammatory rheumatic diseases other than RA: 13 had arthritis associated with skin psoriasis (present at inclusion in 12), 3 had benign sarcoid arthritis, 3 had spondylarthropathy, and 1 had scleroderma. Autoantibodies were present at low frequency in these patients: RF in four, anti-Sa in two, and anti-CCP in one. At each of the follow-up visits, more than two-thirds of the patients (namely 101 of 156 and 101 of 149 at 18 and 30 months, respectively) did not fulfill criteria for a specific diagnosis and might thus be classified as undifferentiated arthritis.

### Autoantibodies as predictors of poor outcomes

We first determined the sensitivity, specificity and positive LR of the presence of autoantibodies at onset as prognostic markers for predefined outcomes (Table 3). Anti-Sa stood out as the single moderately good marker (positive LR 2.16) to predict the development of severe arthritis. In this regard, anti-CCP (positive LR 1.38) and RF (positive LR 1.50) gave similarly poor results. It is remarkable that, despite the lower prevalence of anti-Sa in our cohort, its sensitivity for the severity outcome was very similar to that of RF and anti-CCP. Any combination of RF and/or anti-CCP with anti-Sa was no better than anti-Sa alone in predicting severe outcomes. In contrast, antibodies present at inclusion were specific but poorly sensitive predictors of persistent arthritis at 30 months. Owing to our inclusive *a priori *definition (see above and similar to that in [25]), persistent arthritis was observed in 84% of the patients at 30 months. Thus, despite their strong positive LR, antibodies were not clinically helpful in predicting persistence in our cohort at the 30-month evaluation mark. We therefore looked at alternative definitions of persistent arthritis, such as non-remission defined by DAS28 (namely presenting a DAS28-3 score of at least 2.6). Non-remission defined by DAS28-3 was present in 45% of our aggressively DMARD-treated patients at the 30-month evaluation. None of the three antibodies was correlated with non-remission defined by DAS-28 (data not shown), suggesting that the presence of antibodies does not correlate with a poorer response to (conventional) DMARD treatments. Persistence defined as non-remission defined by DAS28-3 was therefore not used further. Finally, no autoantibody correlated with the fulfillment of RA criteria, mostly because of poor specificity. The best serological markers for the fulfillment of criteria for RA at 30 months were RF and anti-Sa, which gave low but very similar positive LR values (1.53 and 1.54, respectively). This result probably reflects the dampening impact of treatment on the clinical activity of arthritis.

In all three evaluated outcomes, RF had a higher sensitivity than anti-Sa, whereas anti-Sa had a higher specificity than RF (except for the fulfillment of RA criteria). Anti-CCP also had a slightly better specificity than RF for predefined outcomes. However, despite their high specificity for established RA, anti-CCP antibodies performed poorly. The same differences between anti-CCP and anti-Sa antibodies, although present, were less marked at the 18-month evaluation (data not shown). As noted above, anti-Sa alone performed better than any combination of antibodies in predicting severity. The conclusions were not significantly different when the analysis was restricted to patients fulfilling RA criteria at inclusion (data not shown). As noted in Table 2, specific avoidance of DMARDs in anti-Sa patients could be discarded as a possible explanation for their more rapid radiographic deterioration, because these patients were as intensively treated as patients with RF.

### Multivariate prognostic models of severity

As illustrated in the multiple regression model for severity (Table 4, model A), anti-Sa (OR 8.832) clearly outperformed RF and anti-CCP. Indeed, the presence of anti-Sa conferred an even higher OR than erosion SvH abnormalities (OR 3.472), and was the most powerful independent predictive variable for the severity outcome. The OR of anti-Sa increased after adjustment for all other included variables. Although anti-Sa was the best individual marker for severity, a combination of anti-Sa, an SvH erosion score of at least 5 and increasing age best explained severe disease development. Worth noting is the absence of a HLA-DR 'shared-epitope', disability defined by M-HAQ, and the fulfillment of ACR criteria for RA from the short list of variables present at inclusion that were predictive of the predefined severe outcome.

However, anti-Sa antibodies are not widely available yet, and their presence in the model might have wiped out significant associations of RF and/or anti-CCP with severe arthritis. After the removal of anti-Sa from the analysis, neither RF nor anti-CCP was significantly predictive of severe arthritis (Table 4, model B). At that time, we suspected some heterogeneity among patients with anti-CCP. Receiver operating characteristics curves indicated that a cut-off of 61 U/ml of anti-CCP, rather than 21 U/ml, would yield an improved positive LR of 2.01 for the severity outcome (data not shown). Similarly, after deletion of anti-Sa results and restriction to titers of anti-CCP higher than 60 U/ml, the predictive value of anti-CCP improved but remained below that of RF; neither reached a statistically significant level (Table 4, model C).

Finally, we tested the interaction of erosion SvH score and anti-Sa, the two major initial predictors of severe arthritis on the development of joint damage (Fig. 1). Each of the two predictors influenced the 30-month SvH score, with significant erosive damage at inclusion being the most determinant for the rate of progression of radiological damage. In patients in whom erosion SvH scores were abnormal at inclusion, the rate of yearly increase of the total SvH score was about 10 units. This rate was about 4 units/year in those whose initial erosion SvH scores were less than 5 units. These data support the importance of early damage as evidence for aggressive disease.

In the multiple regression model for persistence at 30 months (data not shown), disease duration for at least 4 months at inclusion was the single significant prognostic marker. No antibody was statistically associated with the outcome of persistence at that time.

## Discussion

The main conclusion from our EPA study is that assays targeting different citrullinated antigens have distinct prognostic values for poor outcomes, in addition to their differences in sensitivity and specificity [20]. When present, anti-Sa antibodies are useful markers of poor prognosis in EPA patients, even when rapidly treated intensively with conventional DMARDs. In our EPA population, anti-Sa outperformed anti-CCP antibodies for the prediction of each of the predetermined clinical outcomes, at least up to 30 months into disease. Despite their more specific association with RA [20,46], anti-CCP antibodies did not carry any significant advantage over RF. It might thus be premature to consider replacing RF by anti-CCP antibodies in the clinical evaluation of EPA patients [47]. Differences between RF and anti-Sa antibodies were also significant. As a rule, RF proved to be the most sensitive assay for all outcomes, whereas anti-Sa was the most specific.

At first sight, the lower prevalence of anti-Sa (28% versus 41% for RF) seemed a significant disadvantage. However, the sensitivity of anti-Sa for severe arthritis increased over time in our cohort, as 29%, 39%, and 45% of all severe patients had anti-Sa at inclusion, 18 months, and 30 months, respectively. This observation held true even though anti-Sa positive patients received as aggressive DMARD treatments as the other patients with persistent disease. The lack of statistically significant association of antibodies with persistence was probably due in part to the dual definition of persistence that we used, namely the presence of synovitis and/or being treated. In the absence of a more specific diagnostic test for persistence, we consider this definition to be the best currently available in treated recent-onset arthritis. The major limitation with our definition of persistence was the unexpectedly high (80%) frequency of ongoing use of DMARDs at 30 months. We expect that most patients in protracted remission will slowly taper their DMARDs over the planned follow-up of up to 5 years. The confounding influence of prolonged treatment on our definition of persistence should thus progressively attenuate over these additional 30 months, and the prognostic value of antibodies for persistence might then become assessable.

The second important conclusion is that autoantibodies were present early in only 50 to 60% of the patients who developed any of the predefined poor outcomes at 30 months. Autoantibodies present early into disease therefore characterize a large, but limited, subset of EPA patients with poor outcomes. Detection of autoantibodies in EPA patients must thus be used in combination with additional variables present at inclusion (for example elevated erosion SvH scores and increasing age) to best predict, and possibly prevent, the development of severe disease at 30 months (and beyond).

The third conclusion is that our results should be generally applicable to EPA patients evaluated by rheumatologists. Several reasons support that opinion. First, selection biases were minimized by the strict exclusion of patients with diseases known to have a good prognosis (such as crystal-induced arthritis and monoarthritis) and by a retention rate of 90% at 30 months, without individual missing data. Second, the patients were thoroughly evaluated with widely available tools (except for anti-Sa), early in their disease (median 3 months), and at consistent intervals relative to disease onset. Third, the predefined severity outcome included both functional disability and radiographic damage. Functional impairment and invalidity are the best indicators of severity, but they occur too late into disease to be useful prognostic indicators. Radiographic changes, such as those quantified with the SvH score, are therefore used as surrogates of severity in early disease. Because disability and radiographic damage are poorly correlated in early arthritis [45,48], both contribute in relatively independent ways to the full picture of severity during the first years of chronic arthritis.

Our observations regarding the prognostic usefulness of anti-CCP in EPA patients are somewhat at variance with other reports. The reasons for differences in prognostic value between anti-CCP and anti-Sa assays therefore merit further discussion. A first possible explanation relates to deficiencies of the specific commercial anti-CCP assay used in this paper. That explanation is unlikely. We observed no significant differences in sensitivities or specificities between commercial anti-CCP assays (G. Boire, unpublished observations). Such differences would be unlikely, because all commercially available anti-CCP assays use the same antigen strips [20] and differ only in technical aspects of the ELISA itself. Thus, the prognostic performance of the particular anti-CCP assay we used is likely to be generally applicable to commercial anti-CCP assays.

A second and more likely possibility for the poor performance of anti-CCP might reside in the design of the assay. The objectives of current anti-CCP assays are to attain the maximal sensitivity for patients with established RA or RA-like disease, while maintaining a reasonable specificity. This approach favors a low threshold to report positive results. Because EPA and RA are very heterogeneous diseases, a more appropriate design would be to identify subsets of patients with worse outcomes. In our cohort, most anti-CCP positive sera had high titers (mean 135.6 U/ml; median 116 U/ml) but, significantly, 11 patients had low or moderate titers (60 U/ml and below). The presence of low or moderate titers of anti-CCP at inclusion did not seem to be associated with severe arthritis at 30 months. Indeed, using a cut-off of 61 U/ml afforded the best OR estimate for the development of severe arthritis, although the predictive value of this level of anti-CCP was still not statistically significant (Table 4, model C). It was previously suggested that many non-RA sera with low anti-CCP titers bind similarly to citrullinated and non-modified peptides [49]. These sera would present as false positive anti-CCP results. As a consequence, we suggest that the threshold for positive results of commercial anti-CCP assays should be adjusted upward to increase their prognostic value in EPA patients.

A third explanation for the prognostic discrepancy between anti-CCP and anti-Sa implies hypothetical qualitative differences between the anti-Sa and the anti-CCP assays, as suggested by the moderate degree of association between anti-Sa and anti-CCP results remaining constant across different cut-offs for positive anti-CCP (phi coefficient 0.6881 for titers higher than 21 U/ml, and 0.6339 for titers higher than 100 U/ml). These qualitative differences between assays would translate into genuine consequent associations with outcomes. Antibodies present in sera from RA patients recognize citrullinated peptidic residues in the context of adjoining amino acid residues and of peptide conformation [19,20]. The short synthetic peptides used in anti-CCP ELISA were circularized and covalently bound to plastic strips to circumvent some of the problems inherent to the use of peptides in solid-phase assays [46]. This design might not be appropriate in very early polyarthritis, at a time when the immune response to citrullinated antigens is still being matured *in vivo *by modified arginine-rich proteins. At present it remains to be confirmed whether citrullinated proteins in general might perform better than CCP in ELISA at identifying severe subsets of EPA patients.

A fourth contributing factor to explain the poor performance of anti-CCP in our cohort is the influence of aggressive treatment on EPA prognosis. An earlier time of introduction, a higher intensity of use, and a more prolonged maintenance of DMARDs, with the objective of controlling disease, distinguish our current cohort from previous EPA cohorts established during the early 1990s. This more aggressive approach is illustrated by the high prevalence of DMARDs still used at 30 months, as well as by the marked decrease in disease activity over time, as assessed by the DAS28-3, the swollen joint counts, and the M-HAQ. Possibly because of this relatively intensive use of DMARDs, more than half of the patients were in clinical remission at 30 months. According to this hypothesis, the presence of anti-CCP antibodies would indeed identify patients with a poor natural course, as suggested in previous EPA or early RA cohorts [25-27,50]. In contrast, anti-CCP would fail to identify patients who do poorly when exposed relatively early to intensive treatment with current DMARDs. Such an effect of aggressive treatment was previously reported to explain the loss of HLA-DR association with severity in aggressively DMARD-treated patients [51]. If this is true for anti-CCP, however, patients with anti-Sa would not exhibit the same generally good response to DMARDs or, alternatively, the association of anti-Sa with severe outcomes would be so large that the use of DMARDs would not completely erase it. Because intensive and effective DMARD treatments capable of slowing the progression of radiological and functional deterioration are increasingly being used in clinical practice, progressively longer periods of observation in larger samples of patients should therefore be required to delineate the true predictive value of potential prognostic markers.

Finally, our data stress the importance of intrinsic aggressiveness of arthritis in causing progressive joint damage. At inclusion, 18% of the patients already demonstrated clinically significant erosive changes. That represents a very early progression to the pannus phase, and an already missed opportunity to intervene during the optimal 'therapeutic window' in these patients. Severe outcomes at 30 months happened mostly in those patients who already had significant erosive joint damage at first evaluation. Early erosive changes are therefore an excellent surrogate marker for an aggressive arthritis. Whether the use of more sensitive imaging techniques such as ultrasound or magnetic resonance imaging [52] would increase the sensitivity of detection without affecting its specificity for poor outcomes remains to be defined [52-54]. In contrast, a serological marker such as anti-Sa antibodies, more highly associated with severe outcomes than RF or anti-CCP, and present at inclusion in a patient without detectable erosions, would probably represent a pre-pannus surrogate marker for aggressive arthritis. Such a marker would be extremely useful in clinical trials and in real-world practice as well, to select patients for appropriate treatment. In that situation, additional independent markers (for example inflammatory markers, possibly genetic markers) will still be needed if we are to attain a more complete prediction of the outcomes in individual EPA patients.

## Conclusion

This study reports the first direct comparison of RF, anti-CCP and anti-Sa antibodies as prognostic markers in a cohort of patients with recent-onset inflammatory polyarthritis treated according to current standards. In this cohort, anti-Sa antibodies were present at inclusion in 45% of the patients who subsequently developed severe outcomes at 30 months. Anti-Sa antibodies were the single serological marker independently predictive of poor outcomes and, together with the presence of joint erosions at inclusion and with increasing age, characterized a subgroup of patients with more rapid and more severe joint damage. In contrast, anti-CCP antibodies were not independently associated with severe outcomes in our patients. Using higher thresholds for positive results slightly improved the performance of the anti-CCP assay, but the predictive value of anti-CCP antibodies remained inferior to that of RF. Although anti-Sa and anti-CCP assays both use citrullinated antigens, our data suggest that the two assays differ in their ability to predict poor outcomes in patients treated aggressively early after the onset of inflammatory polyarthritis.

## Abbreviations

ACR = American College of Rheumatology; BSA = bovine serum albumin; CCP = cyclic citrullinated peptide; CHUS = Centre hospitalier universitaire de Sherbrooke; CRP = C-reactive protein; DAS28-3 = Disease Activity Score using 28 joints and 3 variables (tender and swollen joint counts and CRP); DMARD = disease-modifying anti-rheumatic drug; ELISA = enzyme-linked immunosorbent assay; EPA = early (or recent-onset) polyarthritis; LR = likelihood ratio; MBP = myelin basic protein; M-HAQ = modified Health Assessment Questionnaire; OR = odds ratio; PADI = peptidylarginine deiminase; PBS = phosphate-buffered saline; RA = rheumatoid arthritis; RF = rheumatoid factor; SvH = Sharp–van der Heijde; WB = western blotting.

## Competing interests

The author(s) declare that they have no competing interests.

## Authors' contributions

GB designed the research protocol, played a leading role in the design and coordination of the study, and drafted the manuscript. NC performed the statistical analysis and helped to draft the manuscript. PC helped in the design of the research protocol, longitudinally contributed to the coordination of the study, and helped in drafting the manuscript. AJF participated in the performance and the coordination of the study, and helped to draft the manuscript. PL participated in the performance and the coordination of the study, and helped to draft the manuscript. TN participated in the design of the protocol, contributed to the coordination of the study, supervised the statistical analysis, and reviewed the draft manuscript. ZJZ developed the anti-Sa assays and performed a large portion of the assays for the study. CD participated in the design of the protocol and performed the immunogenetic tests. HAM designed the research protocol, contributed to the performance of the study, supervised the development and performance of anti-Sa assays, and helped to draft the manuscript. All authors read and approved the final manuscript.

## Figures and Tables

**Table 1:** Baseline characteristics of the study population (*n *= 165)

Characteristic	Value
No. female (%)	95 (57.6)
Median age, years (range)	58.8 (19–85)
Median duration of symptoms, months (range)	3 (1–12)
No. with disease duration of 3 months or less (%)	87 (52.7)
Median number of joints with synovitis (range)	10 (3–58)
No. fulfilling at least four ACR criteria for RA (%)	133 (80.6)
Mean M-HAQ score (range)	0.93 (0–2.75)
No. with M-HAQ score ≥ 1.0 (%)	77 (47.0)
Median DAS28-3 score (range)	5.01 (2.09–7.85)
No. with RF ≥ 40 IU/mL (%)	68 (41.2)
No. with anti-CCP ≥ 20 U/mL; positive (%)	53 (33.1)
No. with positive anti-Sa antibodies (%)	46 (28.0)
No. with CRP ≥ 8.0 mg/L (%)	119 (72.6)
No. with at least one HLA-DR 'shared epitope' (%)	77 (47.0)
No. with two HLA-DR 'shared epitopes' (%)	22 (13.4)
Median SvH score (range)	4 (0–54)
No. with total SvH score ≥ 10 (%)	31 (18.8)
No. with erosion SvH score ≥ 5 (%)	30 (18.2)

ACR, American College of Rheumatology; CCP, cyclic citrullinated peptide; CRP, C-reactive protein; DAS28-3, Disease Activity Score using 28 joints and 3 variables (tender and swollen joint counts and C-reactive protein); M-HAQ, modified Health Assessment Questionnaire; RA, rheumatoid arthritis; RF, rheumatoid factor; SvH, Sharp–van der Heijde.

**Table 2:** Clinical assessments and outcomes at inclusion and during follow-up

Measure	Inclusion (*n *= 165)	18 months (*n *= 156)	30 months (*n *= 149)
Mean M-HAQ score (median)	0.93 ± 0.64 (0.875)	0.42 ± 0.50 (0.25)	0.35 ± 0.41 (0.25)
No. with M-HAQ ≥ 1.0 (%)	76 (46.1)	18 (11.6%)	17 (11.5%)
Mean DAS28-3 score (median)	5.01 ± 1.27 (4.94)	2.94 ± 1.26 (2.66)	2.81 ± 1.06 (2.46)
No. with DAS28-3 < 2.6 (%)	5 (3.0)	76 (48.7)	82 (55.0)
Mean swollen joint count (median)	12.55 ± 9.57 (10)	2.83 ± 5.51 (0)	2.04 ± 3.64 (0)
Number without synovitis (%)	n.a.	72 (46.2)	82 (55.0)
Number with persistent arthritis (%)	n.a.	137 (87.8)	125 (83.9)
No. fulfilling criteria for RA (%)	133 (80.6)	36 (23.1)	28 (18.8)
No. with severe arthritis (%)	106 (64.2)	65 (41.7)	56 (37.8)
Mean total SvH score (median)	6.05 ± 8.38 (4)	11.34 ± 13.82 (7)	15.76 ± 18.38 (10)
Mean erosion SvH score (median)	3.02 ± 4.99 (1)	6.94 ± 9.37 (4)	10.31 ± 13.14 (7)
Upper third of the total SvH score	>5	>10	>15
No. on DMARDs (%)			
Total	31 (18.8)	120 (76.9)	116 (77.9)
RF-positive	13/68 (19.1)	60/63 (95.2)	60/61 (98.4)
Anti-CCP-positive	10/53 (18.9)	49/52 (94.2)	50/51 (98.0)
Anti-Sa-positive	6/46 (13.0)	43/45 (95.6)	44/44 (100.0)

CCP, cyclic citrullinated peptide; DAS28-3, Disease Activity Score using 28 joints and 3 variables (tender and swollen joint counts and C-reactive protein); M-HAQ, modified Health Assessment Questionnaire; n.a., not applicable; RF, rheumatoid factor; SvH, Sharp–van der Heijde. Where errors are shown, results are means ± SD.

**Table 3:** Autoantibodies as prognostic markers of poor outcomes

Autoantibody	Measure	Outcomes at 30 months		
		
		Persistence	RA criteria	Severity
RF	Sensitivity	47.2	57.1	55.4
	Specificity	91.7	62.8	63.0
	Positive LR	5.69	1.53	1.50
Anti-Sa	Sensitivity	34.4	39.3	44.6
	Specificity	95.8	72.7	79.3
	Positive LR	8.26	1.44	2.16
Anti-CCP	Sensitivity	39.2	39.3	42.9
	Specificity	91.7	66.9	70.7
	Positive LR	4.70	1.19	1.46

Combinations of antibodies (rheumatoid factor (RF) and/or anti-Sa and/or anti-cyclic citrullinated peptide (anti-CCP)) are not shown, because none brought any improvement over the detection of anti-Sa alone. LR, likelihood ratio; RA, rheumatoid arthritis.

**Table 4:** Odds ratio estimates of severe arthritis at 30 months from independent variables present at inclusion

Variable at presentation	Odds ratio estimate (95% confidence limits)		
	
	Model A	Model B	Model C
RF ≥ 40 IU/ml	1.788 (0.549–5.825)	2.946 (0.957–9.073)	2.110 (0.715–6.231)
Anti-CCP ≥ 20 U/ml	0.320 (0.075–1.357)	0.917 (0.285–2.953)	-
Anti-Sa-positive	8.832 (2.141–36.436)***	-	-
Anti-CCP ≥ 60 U/ml	-	-	1.693 (0.551–5.201)
Age (per year)	1.063 (1.025–1.102)***	1.049 (1.015–1.084)***	1.048 (1.014–1.084)***
Male sex	1.741 (0.718–4.223)	1.627 (0.703–3.767)	1.700 (0.733–3.945)
Disease duration	1.686 (0.701–4.058)	1.647 (0.715–3.793)	1.603 (0.700–3.673)
Morning stiffness	0.415 (0.154–1.118)	0.554 (0.218–1.406)	0.502 (0.196–1.284)
Symmetry of arthritis	0.798 (0.174–3.657)	1.085 (0.241–4.880)	1.315 (0.282–6.124)
Swollen joint count	0.985 (0.940–1.033)	0.989 (0.946–1.033)	0.988 (0.945–1.033)
M-HAQ ≥ 1.0	0.666 (0.266–1.666)	0.750 (0.315–1.789)	0.791 (0.330–1.897)
CRP ≥ 8 mg/L	2.594 (0.920–7.312)	2.107 (0.795–5.581)	2.139 (0.807–5.667)
HLA shared epitope	1.096 (0.444–2.704)	0.982 (0.417–2.313)	0.937 (0.401–2.186)
Erosion SvH score	3.472 (1.236–9.575)*	3.751 (1.388–10.135)*	3.611 (1.340–9.729)**

Multivariate regression analysis was performed using all three autoantibodies (model A), without anti-Sa (model B), and without anti-Sa but with a higher threshold of 61 U/ml for anti-cyclic citrullinated peptide (anti-CCP) (model C). Confidence limits are 95% Wald confidence limits. Disease duration was set as positive when present for at least 4 months. Morning stiffness was set as positive when it lasted for at least 60 min. The swollen joint count represents the contribution of each additional joint above the inclusion requirement of three. HLA shared epitope indicates the presence of at least one of the HLA DR alleles associated with RA, as listed in the Methods section. Erosion Sharp–van der Heijde (SvH) score was set as positive when the value was 5 or more. CRP, C-reactive protein; M-HAQ, modified Health Assessment Questionnaire; RF, rheumatoid factor. *P < 0.02; **P < 0.01; ***P < 0.005.
